# Changes in the mechanical properties of tendon structures in lower limbs during repetitive contractions in male long‐distance runners

**DOI:** 10.14814/phy2.70859

**Published:** 2026-03-31

**Authors:** Shuhei Sasajima, Daisuke Miyazaki, Shigeharu Tanaka, Keitaro Kubo

**Affiliations:** ^1^ Department of Life Science The University of Tokyo Meguro Tokyo Japan; ^2^ Department of Physical Education Kokushikan University Setagaya‐ku Tokyo Japan

**Keywords:** elongation, hysteresis, medial gastrocnemius muscle, ultrasonography, vastus lateralis muscle

## Abstract

The purposes of this study were to compare mechanical properties of tendon structures during single and repetitive contractions between long‐distance runners (LDR) and untrained males (UTM) and to verify the relationship between changes in mechanical properties of tendon structures during repetitive contractions and running performance in LDR. Mechanical properties of tendon structures of vastus lateralis (VL) and medial gastrocnemius (MG) muscles were measured during single contraction. In addition, changes in mechanical properties of VL and MG tendon structures were measured during 20 min of repetitive contractions. During single contraction, maximal elongation of tendon structures was significantly lower in LDR than in UTM for VL, but not MG. During repetitive contractions, hysteresis of VL and MG tendon structures increased significantly for both groups. In LDR, the relative change in hysteresis of MG tendon structures, but not VL, during repetitive contractions was significantly correlated with the best official record in the 5000‐m race. In conclusion, LDR exhibited lower extensibility of tendon structures for VL compared to UTM, while both LDR and UTM showed significant increases in hysteresis of VL and MG tendon structures during repetitive contractions. Furthermore, LDR with higher competitive performance showed a lower increase in hysteresis of tendon structures for MG during repetitive contractions.

## 1 INTRODUCTION

Numerous studies have demonstrated that the mechanical properties of tendons contribute to performance and efficiency during stretch‐shortening cycle exercises, for example, running and jumping (Fukunaga et al., 2002; Lai et al., 2014; Lichtwark et al., 2007). Among various sports competitions, long‐distance running is a typical stretch‐shortening cycle exercise. Over the past 20 years, previous studies using ultrasonography have revealed that during the contact phase of running, the whole muscle‐tendon unit length exhibits a stretch‐shortening cycle, while fascicles continue to shorten (Bohm et al., 2019; Ishikawa & Komi, 2008). Furthermore, several previous studies reported that performance in long‐distance running (e.g., the best official record in a 5000 m race) and running economy (defined as the steady‐state oxygen consumption for a given running velocity; e.g., Fletcher et al., 2009) were associated with tendon mechanical properties in the lower limbs (Albracht & Arampatzis, 2013; Fletcher et al., 2010; Kubo et al., 2010, 2015). However, the findings of these previous studies were not consistent. Some studies demonstrated that long‐distance runners with compliant tendon mechanical properties in lower limbs exhibited superior performance and economy of running (Arampatzis et al., 2006; Kubo et al., 2010, 2015). Conversely, other studies reported that improving running economy could be achieved by increasing Achilles tendon stiffness through isometric training (Albracht & Arampatzis, 2013; Fletcher et al., 2010). On the other hand, in these previous studies, tendon mechanical properties were measured at a “low strain rate” and under “single‐contraction” conditions. However, the conditions of tendon elongation during long‐distance running (i.e., high strain rate and repetitive contractions) differ considerably from the conditions used to measure tendon mechanical properties in previous studies cited above.

Regarding the difference in the strain rate of tendon, our recent studies demonstrated that tendon mechanical properties measured under high strain rate (i.e., ballistic contractions) exhibited smaller elongation and greater hysteresis compared to those measured under low strain rate (i.e., ramp contractions; commonly used in many previous studies) (e.g., Kouno et al., 2021). In addition, previous studies on the mechanical properties of tendons in long‐distance runners have primarily focused on tendon stiffness (Arampatzis et al., 2006; Fletcher & Macintosh, 2015; Kubo et al., 2000, 2010, 2015; Machad et al., 2022; Rosager et al., 2002). On the other hand, to our knowledge, only two studies have examined tendon hysteresis in long‐distance runners during ramp contractions (Wiesinger et al., 2017; Zhang et al., 2023). Therefore, it is necessary to evaluate the tendon mechanical properties of long‐distance runners under high strain rate (i.e., ballistic contractions) that closely resemble the strain rate of the tendon during running. Because long‐distance runners possess high running economy (Morgan et al., 1995; Saunders et al., 2004), the tendons of long‐distance runners may exhibit greater elongation and lower hysteresis under high strain rate conditions compared to those of untrained individuals.

Unlike the single‐contraction condition adopted in many previous studies, tendon mechanical properties under repeated contractions have primarily been measured before and after long‐distance running (e.g., Peltonen et al., 2012). According to the findings of most previous studies (Ackermans et al., 2016; Farris et al., 2012; Lichtwark et al., 2013; Peltonen et al., 2012), the Achilles tendon stiffness did not change following prolonged submaximal running. However, it cannot be denied that tendon mechanical properties may have recovered between the end of running and the start of post‐measurement. Indeed, in vitro studies using animal and human cadaver tendons showed that tendon elongation increased (i.e., creep) and tendon hysteresis decreased during repeated cycles of tensile loading (De Zee et al., 2000; Rigby, 1964; Schatzmann et al., 1998). Accordingly, tendon mechanical properties may change during repeated muscle contractions simulating long‐distance running, and thus these changes may be particularly pronounced in long‐distance runners (especially those with higher competitive performance) since long‐distance runners have higher running economy than untrained individuals (Morgan et al., 1995; Saunders et al., 2004).

The aims of the present study were (1) to compare tendon mechanical properties under high strain rate of tendon (ballistic contractions) between long‐distance runners and untrained individuals, (2) to compare changes in tendon mechanical properties during repetitive contractions simulating long‐distance running between the two groups, and (3) to verify the relationship between the changes described in the second aim and running performance (best official record in a 5000 m race) in long‐distance runners. We hypothesized that (1) long‐distance runners exhibited greater tendon elongation and smaller tendon hysteresis during ballistic contractions compared to untrained individuals, (2) the increase in tendon elongation and decrease in tendon hysteresis due to repetitive contractions are more pronounced in long‐distance runners than in untrained individuals, and (3) the changes in tendon mechanical properties described in the second hypothesis are more pronounced in runners with higher competitive performance.

## 2 METHODS

### 2.1 Participants

The participants for this study were 15 well‐trained male long‐distance runners (LDR) and 15 untrained males (UTM). The physical characteristics of the participants are summarized in Table 1. Age and body mass were significantly lower in LDR than in UTM, whereas height did not differ between the two groups. The duration of training experience in LDR was over 6 years. For LDR, the best official record in a 5000 m race within 1 year before these tests ranged from 14:14 to 14:56 (min:s). All of the UTM were either sedentary or moderately active, but none had participated in any regular exercise program for at least 1 year before the test. This study conformed to the Declaration of Helsinki and was approved by the University of Tokyo's Ethics Committee for Human Experiments, Department of Life Science (Sports Sciences). Participants were thoroughly informed about the procedures to be used and the purpose of this study. All participants provided written informed consent.

**TABLE 1:** Age and physical characteristics of the participants Mean (SD).

	LDR	UTM	*p* and *d* values
Age (years)	19.6 (0.5)	26.1 (4.5)	*p* < 0.001, *d* = 2.054
Height (cm)	170.3 (5.8)	174.3 (6.7)	*p* = 0.091, *d* = 0.639
Body mass (kg)	55.7 (6.4)	73.3 (8.6)	*p* < 0.001, *d* = 2.309

Abbreviations: LDR, long distance runners; UTM, untrained men.

### 2.2 Experimental design

Participants visited the laboratory on three separate occasions. During the first visit, they were familiarized with the laboratory equipment and protocols used in this study. After that, they performed the following two tests on two separate days with at least 2 days between sessions: (1) the measurements of mechanical properties of tendon structures during single contraction (experiment‐1) and (2) the measurement of changes in mechanical properties of tendon structures during 20 min of repetitive contractions (experiment‐2). All measurements were taken on the right leg. For each participant, the order of the two experimental conditions was randomly assigned.

### 2.3 Mechanical properties of tendon structures during single contraction (experiment‐1)

Maximal voluntary isometric contraction (MVC) for knee extension and plantar flexion was measured using specially designed dynamometers (Applied Office, Tokyo, Japan). In the present study, the measurements of mechanical properties of tendon structures were performed at two different strain rates: ramp and ballistic contractions (e.g., Kouno et al., 2021). At low strain rate conditions (i.e., ramp contractions), participants were told to gradually increase torque from a relaxed condition to MVC over approximately 5 s, followed by a 5 s relaxation. At high strain rate conditions (i.e., ballistic contractions), they were told to contract as hard and fast as possible, followed by rapid relaxation. Following a normal warm‐up (e.g., stretching of the main muscle groups), they practiced several submaximal isometric ramp and ballistic contractions to become familiar with each test procedure. The ramp and ballistic contraction tasks were repeated twice for each participant, with a minimum of 2 min between tests. The measured values presented below were the averages of two tests. To minimize systematic effects, the task order (ramp and ballistic contractions) was randomly assigned. Torque signals were amplified and sampled at 1 kHz with a 16‐bit A/D converter (PowerLab/16SP, AD Instruments, Australia). During the knee extension task, adjustable lap belts were used to keep the hips and back securely in place. The ankle was firmly strapped to the lever arm of the dynamometer and fixed with the knee joint flexed at 90 deg. (full extension = 0 deg). During the plantar flexion task, participants lay prone on a test bench, with adjustable lap belts holding their waist and shoulders in place. They were asked to clasp their hands behind their back to avoid pushing with any part of their body other than the plantar flexors. The ankle joint was adjusted to 90 deg. with the knee fully extended, and the foot was securely attached to a footplate mounted on the lever arm of the dynamometer.

Elongation of tendon structures (including outer tendon and aponeurosis) for the vastus lateralis (VL) and medial gastrocnemius (MG) muscles was measured during isometric contractions. In the present study, we focused not on the outer tendons (e.g., patellar tendon), but on the entire tendon structures, including the aponeurosis. We will discuss this point later. The VL and MG muscles were imaged longitudinally with ultrasonic equipment (Prosound α7, Hitachi Aloka Medical, Tokyo, Japan) following previously published protocols (e.g., Kubo et al., 2015). Measurements were taken at two sites: at the proximal 50% of the thigh length for VL and at the proximal 30% of the lower leg length for MG. Ultrasonic images were obtained at these levels by selecting sites that were one‐half of the mediolateral widths of each muscle. An electronic linear array probe (UST 5712, Hitachi Aloka Medical, Tokyo, Japan) was affixed to the dermal surface with adhesive tape to prevent it from sliding. Ultrasonic images were captured on a personal computer at 60 Hz and synchronized with clock timer recordings for further analysis. The tester visually verified the echoes from the aponeurosis and fascicles. Ultrasound images were used to visualize the site where one fascicle was attached to the aponeurosis. The displacement of this point is thought to reflect elongation of tendon structures (Kubo et al., 2015).

The displacement of this point will be attributed to both contractile tension and angular rotation, because the knee joint extends slightly and the ankle joint plantar flexes slightly during an “isometric” contraction (e.g., Magnusson et al., 2001). Therefore, to avoid overestimating the elongation of tendon structures during isometric contraction, angular joint rotation must be taken into account. Additional measurements were conducted under passive conditions to adjust for the elongation of tendon structures (e.g., Kubo et al., 2015). To monitor joint angular rotation, an electrical goniometer (Penny & Giles, Biomechanics Ltd., Gwent, UK) was placed on the lateral aspect of each joint. The displacement of each site caused by rotating the knee and ankle from 100 deg. to 80 deg. was digitized from sonographs. In each participant, the displacement of each site derived from ultrasonic images was corrected for the effect of joint rotation.

In the present study, the elongation of tendon structures at MVC was defined as the maximal elongation of tendon structures. In addition, hysteresis of tendon structures was defined as the region within the torque‐tendon elongation loop divided by the area beneath the curve during the ascending phase. The repeatability of measurements of mechanical properties of tendon structures was confirmed in our previous studies (e.g., Kouno et al., 2021; Sasajima & Kubo, 2025).

### 2.4 Changes in mechanical properties of tendon structures during repetitive contractions (experiment‐2)

**FIGURE 1:**
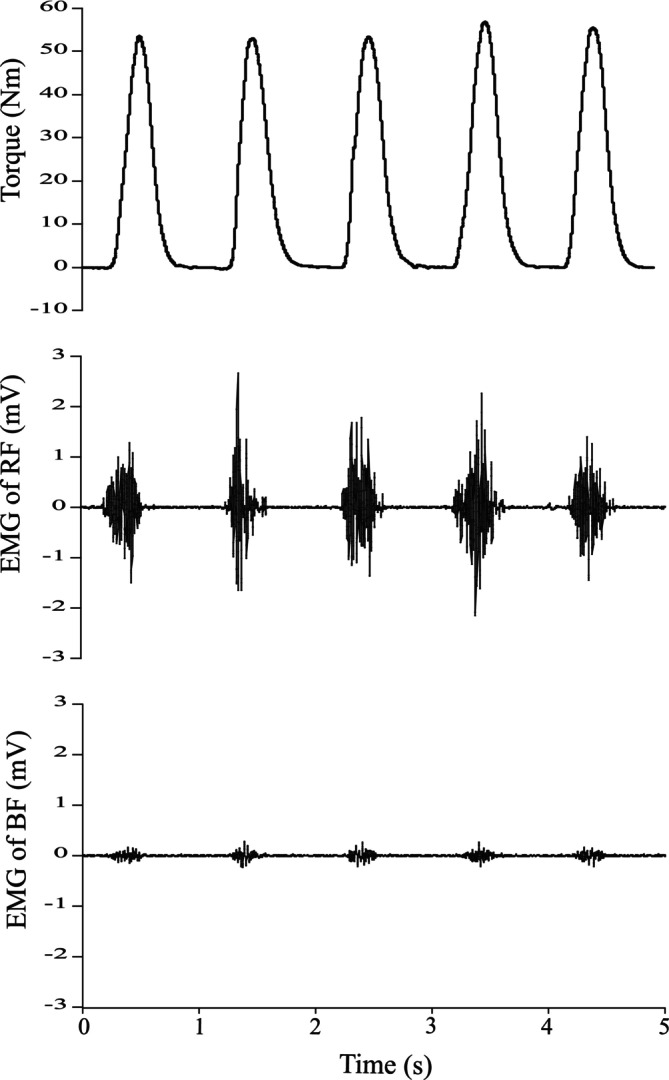
Typical changes in exerted torque during repetitive contractions.

The participant's posture and set‐up were identical to those in experiment 1. The order of knee extension and plantar flexion tasks was randomized for each participant. A 30 min break was taken between the two exercise tasks. After a normal warm‐up, participants performed two or three MVC. The target torque during repetitive contractions was calculated using the highest MVC value. In addition, electromyographic (EMG) activity recorded during MVC measurements was used to normalize EMG data obtained during repetitive contractions (see below). The participant performed 20 min of submaximal repetitive contractions at 50% MVC and 1 Hz (Figure 1). In the present study, the first 20 s (i.e., 0–20 s) and the last 20 s (i.e., 19 min 40 s to 20 min) of 20 min repeated contractions were analyzed. For each of the two analysis time periods, two trials with clear ultrasound images were selected, and the mean values for elongation at peak torque (around 50% of MVC) and hysteresis were calculated. Arampatzis et al. (2023) reported that Achilles tendon elongation during running corresponded to 50% MVC exertion according to the tendon force‐elongation relationship during ramp isometric contraction up to MVC. Therefore, we adopted 50% MVC in experiment‐2 and further stated the limitations of this assumption later. Unfortunately, data for one participant each from the VL tendon structures of LDR, the VL tendon structures of UTM, and the MG tendon structures of UTM were deleted due to poor ultrasonic image quality.

During repetitive contractions, the EMG of the agonist and antagonist muscles was recorded at 1 kHz using a wireless EMG monitoring device (BioLog DL‐5500, S&ME, Japan). Surface electrodes (DL‐510, S&ME, Japan) were attached over the bellies of the rectus femoris (RF), vastus medialis (VM), and biceps femoris (BF) muscles for the knee extension task and the lateral gastrocnemius (LG), soleus (SOL), and tibialis anterior (TA) muscles for the plantar flexion task. The raw data were band‐pass filtered between 20 and 500 Hz. EMG amplitude was rectified and averaged during the ascending and descending phases. mEMG of each muscle was normalized to the maximal effort level.

## 3 STATISTICAL ANALYSIS

Descriptive data represent means ± SD. Differences in the age, physical characteristics, and mechanical properties of tendon structures during single contraction (experiment‐1) were tested using an unpaired Student's *t*‐test. A two‐way analysis of variance (ANOVA) with repeated measures was employed to detect significant effects of group (LDR and UTM) and time (initial and last) on changes in mechanical properties of tendon structures during repetitive contractions (experiment‐2). The F ratios for main effects and interactions were statistically significant at *p* < 0.05. Significant differences among means with *p* < 0.05 were detected using Bonferroni's post hoc test. The homogeneity of variance in an ANOVA was assessed using Mauchly's sphericity test. The Greenhouse–Geisser correction was applied in situations when the sphericity assumption was violated. The effect size was determined using Cohen's *d* formula for an unpaired Student's *t*‐test and partial eta‐squared (*pη* ^*2*^) for two‐way ANOVA. Based on the data distribution, Pearson's or Spearman's correlation coefficients were used to assess the relationships between the changes in mechanical properties of tendon structures during repetitive contractions (experiment‐2) and the best official record in a 5000 m race. The significance level was set to *p* < 0.05.

## 4 RESULTS

Table 2 shows the mechanical properties of tendon structures during single contraction for the two groups. For both knee extensors and plantar flexors, absolute MVC values during ramp and ballistic contractions were significantly lower in LDR than in UTM, but there were no differences in relative MVC values to body mass between the two groups. During ramp and ballistic contractions, maximal elongation of tendon structures was significantly lower in LDR than in UTM for VL, but not for MG. For both VL and MG, no differences in hysteresis of tendon structures during ramp and ballistic contractions were observed between the two groups.

**TABLE 2:** Mechanical properties of tendon structures during single contraction Mean (SD).

		Vastus lateralis muscle		*p* and *d* values	Medial gastrocnemius muscle		*p* and *d* values
		LDR	UTM		LDR	UTM	
Ramp	MVC (Nm)	178.1 (28.5)	222.6 (38.3)	*p* = 0.001, *d* = 1.317	97.4 (17.0)	124.1 (25.8)	*p* = 0.0013, *d* = 1.174
	Relative MVC to body mass (Nm·kg^−1^)	3.2 (0.4)	3.0 (0.4)	*p* = 0.288, *d* = 0.395	1.8 (0.2)	1.7 (0.3)	*p* = 0.429, *d* = 0.293
	Maximal elongation of tendon structures (mm)	16.3 (3.6)	19.6 (4.4)	*p* = 0.032, *d* = 0.825	17.1 (1.9)	18.0 (3.4)	*p* = 0.357, *d* = 0.342
	Hysteresis of tendon structures (%)	20.6 (11.6)	19.8 (17.1)	*p* = 0.886, *d* = 0.053	19.2 (10.4)	14.0 (7.7)	*p* = 0.130, *d* = 0.177
Ballistic	MVC (Nm)	186.1 (30.8)	225.1 (43.9)	*p* = 0.009, *d* = 1.027	97.8 (15.2)	129.3 (26.2)	*p* < 0.001, *d* = 1.471
	Relative MVC to body mass (Nm·kg^−1^)	3.4 (0.5)	3.1 (0.5)	*p* = 0.150, *d* = 0.540	1.8 (0.2)	1.8 (0.3)	*p* = 0.923, *d* = 0.036
	Maximal elongation of tendon structures (mm)	14.3 (3.9)	17.7 (4.3)	*p* = 0.032, *d* = 0.820	15.1 (1.7)	16.9 (4.4)	*p* = 0.143, *d* = 0.550
	Hysteresis of tendon structures (%)	50.5 (9.9)	48.3 (14.9)	*p* = 0.640, *d* = 0.220	52.9 (13.8)	49.5 (17.1)	*p* = 0.553, *d* = 0.219

Abbreviations: LDR, long distance runners; MVC, maximal voluntary contraction; UTM, untrained men.

Tables 3 and 4 show the measured variables during repetitive contractions for knee extension and plantar flexion tasks. All participants sustained the specified torque level (50% of MVC) for 20 min. No differences in peak torque values exerted were observed between the initial and last points analyzed for either group or task. mEMG values of the measured muscles during ascending and descending phases did not change for knee extensors (Table 3). For plantar flexors, mEMG values of other muscles during ascending and descending phases did not change, whereas those for LG during the ascending phase and SOL during the descending phase increased significantly (Table 4).

**TABLE 3:** Changes in the measured variables for knee extensors during repetitive contractions Mean (SD).

	LDR		UTM		Group	*p* and *pη* ^2^ values	Interaction
	Initial	Last	Initial	Last		Time	
Exerted peak torque (Nm)	91.3 (21.5)	95.0 (17.1)	114.1 (25.9)	113.7 (29.7)	*p* = 0.011, *pη* ^*2*^ = 0.225	*p* = 0.325, *pη* ^*2*^ = 0.037	*p* = 0.291, *pη* ^*2*^ = 0.043
Relative mEMG of RF during ascending phase (%)	30.7 (12.0)	32.0 (11.9)	32.8 (11.6)	34.6 (11.2)	*p* = 0.564, *pη* ^*2*^ = 0.012	*p* = 0.321, *pη* ^*2*^ = 0.036	*p* = 0.854, *pη* ^*2*^ = 0.001
Relative mEMG of VM during ascending phase (%)	38.3 (12.2)	36.7 (12.8)	39.0 (8.1)	40.0 (10.6)	*p* = 0.614, *pη* ^*2*^ = 0.010	*p* = 0.791, *pη* ^*2*^ = 0.003	*p* = 0.262, *pη* ^*2*^ = 0.046
Relative mEMG of BF during ascending phase (%)	6.9 (3.8)	6.7 (3.3)	11.3 (9.4)	12.5 (8.9)	*p* = 0.048, *pη* ^*2*^ = 0.138	*p* = 0.463, *pη* ^*2*^ = 0.020	*p* = 0.315, *pη* ^*2*^ = 0.037
Relative mEMG of RF during descending phase (%)	15.5 (6.2)	15.8 (5.4)	14.7 (4.8)	17.3 (7.3)	*p* = 0.841, *pη* ^*2*^ = 0.002	*p* = 0.278, *pη* ^*2*^ = 0.043	*p* = 0.421, *pη* ^*2*^ = 0.024
Relative mEMG of VM during descending phase (%)	13.8 (7.5)	13.7 (7.8)	15.3 (5.6)	16.4 (5.7)	*p* = 0.385, *pη* ^*2*^ = 0.028	*p* = 0.435, *pη* ^*2*^ = 0.023	*p* = 0.399, *pη* ^*2*^ = 0.026
Relative mEMG of BF during descending phase (%)	4.2 (2.8)	3.8 (1.7)	6.0 (4.8)	6.5 (5.4)	*p* = 0.122, *pη* ^*2*^ = 0.086	*p* = 0.845, *pη* ^*2*^ = 0.001	*p* = 0.130, *pη* ^*2*^ = 0.083
Elongation of tendon structures at 50%MVC (mm)	12.9 (1.8)	12.3 (2.4)	13.8 (3.8)	12.8 (3.1)	*p* = 0.485, *pη* ^*2*^ = 0.019	*p* = 0.064, *pη* ^*2*^ = 0.126	*p* = 0.727, *pη* ^*2*^ = 0.005
Hysteresis of tendon structures (%)	42.9 (8.6)	59.4 (8.5)	41.0 (8.4)	60.9 (11.9)	*p* = 0.966, *pη* ^*2*^ = 0.000	*p* < 0.001, *pη* ^*2*^ = 0.773	*p* = 0.383, *pη* ^*2*^ = 0.029

Abbreviations: BF, biceps femoris muscle; LDR, long distance runners; RF, rectus femoris muscle; UTM, untrained men; VM, vastus medialis muscle.

**TABLE 4:** Changes in the measured variables for plantar flexors during repetitive contractions Mean (SD).

	LDR		UTM		Group	*p* and *pη* ^2^ values	Interaction
	Initial	Last	Initial	Last		Time	
Exerted peak torque (Nm)	52.0 (8.3)	49.3 (8.8)	63.9 (14.8)	62.6 (15.1)	*p* = 0.008, *pη* ^*2*^ = 0.235	*p* = 0.052, *pη* ^*2*^ = 0.133	*p* = 0.499, *pη* ^*2*^ = 0.017
Relative mEMG of LG during ascending phase (%)	37.3 (9.9)	43.3 (12.3)	40.9 (14.7)	45.8 (12.3)	*p* = 0.434, *pη* ^*2*^ = 0.023	*p* = 0.037, *pη* ^*2*^ = 0.151	*p* = 0.830, *pη* ^*2*^ = 0.002
Relative mEMG of SOL during ascending phase (%)	48.6 (17.3)	44.5 (11.5)	41.2 (13.9)	42.2 (11.7)	*p* = 0.319, *pη* ^*2*^ = 0.037	*p* = 0.435, *pη* ^*2*^ = 0.023	*p* = 0.217, *pη* ^*2*^ = 0.056
Relative mEMG of TA during ascending phase (%)	4.0 (1.9)	4.0 (1.5)	4.1 (1.7)	4.8 (1.9)	*p* = 0.475, *pη* ^*2*^ = 0.019	*p* = 0.253, *pη* ^*2*^ = 0.048	*p* = 0.260, *pη* ^*2*^ = 0.047
Relative mEMG of LG during descending phase (%)	9.3 (4.4)	11.2 (4.0)	13.2 (6.1)	14.9 (5.9)	*p* = 0.034, *pη* ^*2*^ = 0.156	*p* = 0.050, *pη* ^*2*^ = 0.135	*p* = 0.933, *pη* ^*2*^ = 0.000
Relative mEMG of SOL during descending phase (%)	16.2 (8.1)	18.8 (8.4)	14.2 (4.6)	16.5 (4.9)	*p* = 0.361, *pη* ^*2*^ = 0.031	*p* = 0.024, *pη* ^*2*^ = 0.174	*p* = 0.876, *pη* ^*2*^ = 0.001
Relative mEMG of TA during descending phase (%)	4.0 (4.0)	3.3 (2.8)	2.3 (1.1)	2.6 (1.1)	*p* = 0.185, *pη* ^*2*^ = 0.064	*p* = 0.637, *pη* ^*2*^ = 0.008	*p* = 0.197, *pη* ^*2*^ = 0.061
Elongation of tendon structures at 50%MVC (mm)	12.3 (1.8)	11.7 (1.3)	12.2 (9.9)	9.9 (2.1) ***	*p* = 0.107, *pη* ^*2*^ = 0.093	*p* < 0.001, *pη* ^*2*^ = 0.390	*p* = 0.018, *pη* ^*2*^ = 0.190
Hysteresis of tendon structures (%)	48.5 (10.9)	63.3 (11.8)	45.3 (10.7)	60.4 (11.2)	*p* = 0.406, *pη* ^*2*^ = 0.026	*p* < 0.001, *pη* ^*2*^ = 0.646	*p* = 0.940, *pη* ^*2*^ = 0.000

*Note*: * Significantly different from initial (***p < 0.001).

Abbreviations: LDR, long distance runners; LG, lateral gastrocnemius muscle; SOL, soleus muscle; TA, tibial anteriol muscle; UTM, untrained men.

**FIGURE 2:**
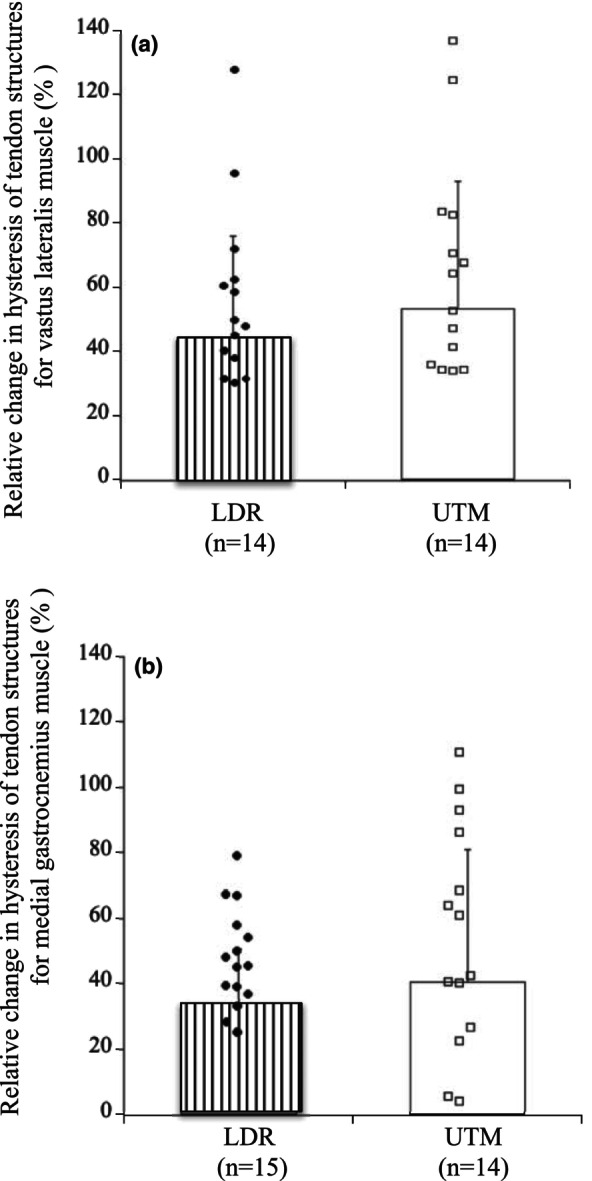
Relative changes in hysteresis of tendon structures during repetitive contractions for vastus lateralis (a) and medial gastrocnemius (b) muscles. LDR; long‐distance runners, UTM; untrained individuals.

Elongation of tendon structures at 50% MVC showed no change for VL of both groups (Table 3), whereas it decreased significantly for MG of UTM but not of LDR (Table 4). In both LDR and UTM, hysteresis of tendon structures for VL and MG increased significantly (Tables 3 and 4). No differences in the relative increases in hysteresis of tendon structures were observed between LDR and UTM for VL (*p* = 0.482, *d* = 0.284; Figure 2A) and MG (*p* = 0.486, *d* = 0.277; Figure 2B).

**FIGURE 3:**
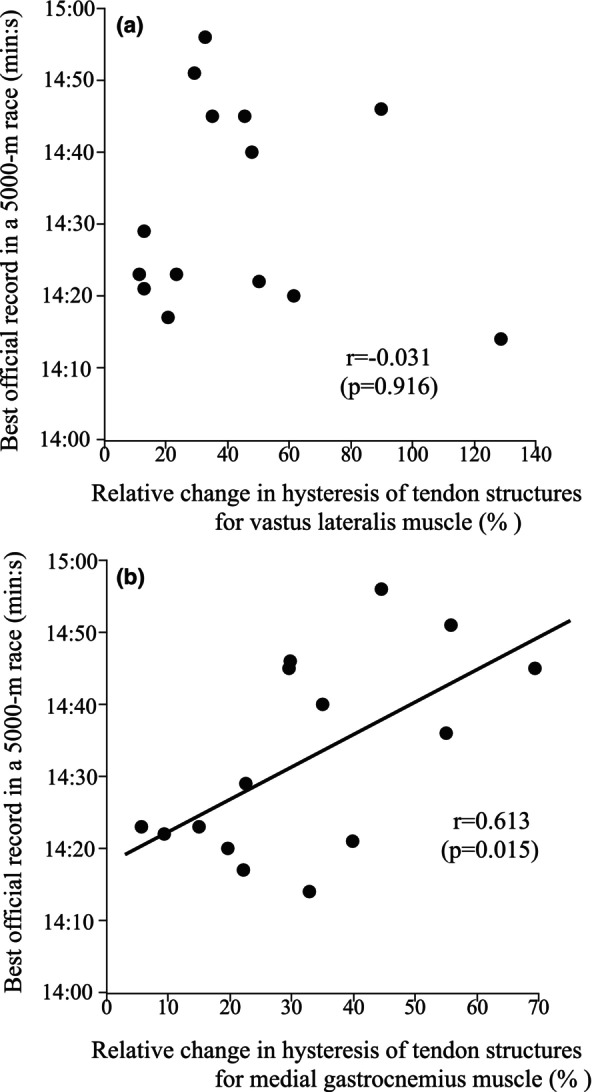
The relationships between the best official record in a 5000 m race and the relative change in hysteresis of tendon structures for vastus lateralis (a) and medial gastrocnemius (b) muscles.

For both VL and MG, the relative changes in elongation of tendon structures at 50% MVC were not significantly correlated with the best official record in a 5000 m race (*r* = 0.074, *p* = 0.793 for VL, *r* = 0.325, *p* = 0.237 for MG). The relative change in hysteresis of tendon structures was significantly correlated with the best official record in a 5000 m race for MG (*r* = 0.613, *p* = 0.015; Figure 3B), but not for VL (*r* = −0.031, *p* = 0.916; Figure 3A).

## 5 DISCUSSION

The main findings of this study were that (1) during single contraction (both ramp and ballistic contractions), maximal elongation of tendon structures for VL (not MG) was significantly lower in LDR than in UTM, whereas no differences in hysteresis of tendon structures of both VL and MG were observed between the two groups, (2) during 20 min of repetitive contractions, hysteresis of tendon structures of both VL and MG increased significantly in both LDR and UTM, whereas no changes in elongation of tendon structures of both VL and MG were observed in either LDR or UTM (except for MG in UTM), and (3) in LDR, runners with higher competitive performance (best official record in a 5000 m race) showed a lower increase in hysteresis of tendon structures for MG, but not VL, during 20 min of repetitive contractions.

During ballistic contractions, maximal elongation of tendon structures of VL, not MG, was significantly lower in LDR than in UTM. These results were consistent with those obtained during ramp contractions in our previous studies (Kubo et al., 2000, 2010). The lower extensibility of tendon structures of VL in LDR compared to UTM is thought to result from the mechanical stress imposed on these tendons by years of extensive running training. Unfortunately, the reason why no such differences were observed in the MG tendons remains unclear. Compared to knee extensors, plantar flexors may have been relatively less affected by the load imposed during running training in LDR, as those are involved in higher levels of activity during daily life activities such as walking (Ericson et al., 1986; Winter & Yack, 1987).

To our knowledge, only two previous studies have examined tendon hysteresis in long‐distance runners (Wiesinger et al., 2017; Zhang et al., 2023). These studies indicated that tendon hysteresis measured during ramp contractions, that is, under low strain rate, was lower in long‐distance runners than in untrained individuals. In the present study, we also examined hysteresis of tendon structures during ballistic contractions and found no difference in hysteresis of tendon structures between the two groups during either ramp or ballistic contractions. The discrepancy between the results of this study and those of the aforementioned previous studies (Wiesinger et al., 2017; Zhang et al., 2023) may be explained by the difference in the measurement sites, that is, outer tendon in previous studies and tendon structures including aponeurosis in this study. In future studies, we need to clarify regional differences in tendon mechanical properties. In addition, no difference was observed between the two groups in hysteresis of tendon structures during repetitive contractions (main effect of group *p* = 0.966 for VL and *p* = 0.406; Tables 3 and 4). Accordingly, it can be concluded that there is no difference in hysteresis of tendon structures between LDR and UTM.

Contrary to our hypothesis, this study found no change in elongation of tendon structures in either LDR or UTM during 20 min of repetitive contractions, except for the MG tendon in UTM. Regarding the MG tendon in UTM, the contribution of the MG to the plantar flexors may have decreased during 20 min of repetitive contractions, since the relative mEMG of LG during the ascending phase increased significantly (Table 4). Our previous studies demonstrated that repeated ballistic contractions simulating running did not increase elongation of tendon structures, whereas elongation of tendon structures significantly increased after repeated isometric contractions of long duration (Kubo et al., 2001, 2005). Kay and Blazevich (2009) and Merza et al. (2022) also reported significant increases in tendon elongation and decreases in tendon stiffness following prolonged‐duration isometric contractions. Therefore, the results of this study (tendon creep did not occur with repeated cyclic contractions) were consistent with the results of these previous studies cited above.

On the other hand, in vitro studies involving multiple‐cycle tensile tests demonstrated that tendons exhibited creep, that is, an increase in elongation of tendon structures (Rigby, 1964; Schatzmann et al., 1998). However, most previous studies examining changes in the mechanical properties of human tendons after running have not confirmed tendon creep (Ackermans et al., 2016; Farris et al., 2012; Lichtwark et al., 2013; Peltonen et al., 2012). The only study involving highly trained long‐distance runners (running more than 100 km per week) found a significant decrease in Achilles tendon stiffness after 90 min of running (Fletcher & Macintosh, 2018). However, according to the tendon force–elongation curves (Figure 3 in Fletcher & Macintosh, 2018), no significant differences were observed in tendon elongation at the same force levels. Therefore, considering the results of these previous and present studies, it is likely that, under cyclic contractions such as running, tendon creep does not occur within the range of durations that humans can sustain. However, it should be noted that the number of contractions in this study (approximately 1200) was considerably lower than the number of contractions (i.e., ground contact) during 90 min of running (approximately 7500) adopted in Fletcher and Macintosh (2018), where tendon stiffness was reduced. Future research should verify this in longer exercise tasks, such as a marathon.

An interesting finding of this study was that hysteresis of tendon structures increased significantly with 20 min of repetitive contractions. To date, no studies have examined the effects of repetitive contractions on the hysteresis of human tendons in vivo. In vitro studies using animal and human cadaver tendons reported that tendon hysteresis decreased during repeated cycles of tensile loading (De Zee et al., 2000; Rigby, 1964; Schatzmann et al., 1998). Based on these in vitro findings, we predicted that repetitive contractions would reduce hysteresis of tendon structures, with this reduction particularly pronounced in long‐distance runners with high running economy. However, contrary to these predictions, hysteresis of tendon structures increased significantly with repeated contractions, and no difference in the increase of hysteresis of tendon structures was observed between LDR and UTM. Increased hysteresis of tendon structures indicates increased viscosity within the tendons. Furthermore, these results may also reflect changes in the mechanical properties of aponeurosis, as this study examined the entire tendon structures, including the aponeurosis. On the other hand, it is also possible that the hysteresis of tendon structures measured in this study increased due to the delayed fascicle elongation upon relaxation caused by fatigue of fascicles (i.e., due to increased hysteresis of the fascicle). Unfortunately, the measurement method adopted in this study makes it impossible to determine whether the increase in tendon hysteresis associated with repeated contractions was caused by a change (increase) in the viscosity of either the tendon or the fascicle tissue. Future research must develop a method to separately quantify the viscosity of human tendons and fascicles to clarify this point.

The other significant finding of the present study was that long‐distance runners with superior competitive performance (best official record in a 5000 m race) exhibited a lower increase in hysteresis of tendon structures of MG, but not VL, due to repetitive contractions. This result suggested that suppressing the increase in hysteresis of tendon structures of MG during long‐distance running improved athletic performance. According to our previous study (Kubo et al., 2015), among a large group of long‐distance runners (*n* = 64), a significant correlation was observed between the best official record in a 5000 m race and the stiffness of MG tendon structures. Therefore, the results of this study on the hysteresis of tendon structures partially align with those on the stiffness of tendon structures. Considering our previous and present findings, it may be fair to say that the mechanical properties (elasticity and viscosity) of the MG tendon structures, rather than the VL tendon structures, significantly influence athletic performance for long‐distance runners.

There were several limitations in the present study. Firstly, the mechanical properties of tendon structures were measured during isometric contractions, not during running. Several studies quantified tendon mechanical properties by measuring changes in fascicle length during stretch‐shortening cycle exercises (e.g., running and jumping; Farris et al., 2011; Lichtwark & Wilson, 2005), but the calculated tendon elongation values relied on estimates derived from whole‐muscle‐tendon complex length calculated from changes in joint angles. Therefore, measurements taken during isometric contractions were considered more accurate for assessing elongation of tendon structures than those taken during jumping or running. In the present study, the mechanical properties of tendon structures were measured not only at a low strain rate (ramp contractions), as adopted in many previous studies, but also at a high strain rate (ballistic contractions). Secondly, the conditions of experiment‐2 were not strictly the same as those of long‐distance running. In this study, experiment‐2 was set to 20 min, simulating a 5000 m race. In addition, the force level was set at 50% MVC based on the results of previous study that estimated muscle force from tendon elongation during running (Arampatzis et al., 2023). However, the fascicle dynamics and tendon strain rate during repeated isometric contractions in experiment‐2 cannot be considered strictly identical to those observed during long‐distance running. Therefore, in the future, it will be necessary to conduct actual measurements of changes in the mechanical properties of tendons during long‐distance running. Thirdly, we investigated the mechanical properties of whole tendons (including the outer tendon and aponeurosis), but not those of the outer tendon alone. Previous studies reported that the mechanical properties of the outer tendon differ from those of the aponeurosis (Magnusson et al., 2003; Stafilidis et al., 2005). In addition, recent studies have demonstrated that the roles of aponeurosis in force transmission in musculoskeletal function are more complex due to variations in microstructural collagen fibril alignment (Grega et al., 2020; Wheatley et al., 2023). The outer tendon and aponeurosis may respond differently to repetitive contractions. Indeed, Lichtwark et al. (2013) reported that the strain of the outer tendon (calcaneus to soleus) significantly increased after a 5 km run, whereas that of the gastrocnemius tendon (calcaneus to gastrocnemius) did not. Although this has not yet been demonstrated experimentally, it is possible that changes in fascicle length (and muscle function) are more closely associated with the mechanical properties of tendon structures (including aponeurosis) than with those of outer tendons. Fourthly, all participants in the present study were males. Previous studies demonstrated that differences in tendon stiffness and hysteresis were observed between males and females (Gianakos et al., 2024; Onambele et al., 2007). Therefore, there may be sex differences in the changes in tendon mechanical properties during repetitive contractions between males and females. However, because the menstrual cycle in females may alter the outcomes (Eiling et al., 2007; Sarwar et al., 1996), only males were included in this investigation.

The present results demonstrated that male long‐distance runners exhibited lower extensibility of tendon structures in VL, but not in MG, compared to untrained males, while both groups showed significant increases in hysteresis of tendon structures of VL and MG following repeated contractions. Furthermore, long‐distance runners with higher competitive performance showed a lower increase in hysteresis of MG tendon structures during 20 min of repetitive contractions. Our previous study showed that the stiffness of MG tendon structures was significantly correlated with the best official record in a 5000 m race (7). These results implied that the mechanical properties (elasticity and viscosity) of the MG tendon structures have a considerable impact on long‐distance running performance.

## AUTHOR CONTRIBUTIONS

**Shuhei Sasajima:** Formal analysis; investigation; resources. **Daisuke Miyazaki:** Formal analysis; investigation. **Shigeharu Tanaka:** Conceptualization; methodology; supervision. **Keitaro Kubo:** Conceptualization; methodology; project administration; supervision.

## FUNDING INFORMATION

This work was supported by JST SPRING, Grant Number JPMJSP2108.

## CONFLICT OF INTEREST STATEMENT

The authors declare no conflicts of interest.

## Data Availability

All data supporting the results are presented in the manuscript.
